# Parents' perception of children's fear: from FSSC-IT to FSSC-PP

**DOI:** 10.3389/fpsyg.2015.01199

**Published:** 2015-08-12

**Authors:** Silvia Salcuni, Carla Dazzi, Stefania Mannarini, Daniela Di Riso, Elisa Delvecchio

**Affiliations:** ^1^Dipartimento di Psicologia dello Sviluppo e della SocializzazionePadova, Italia; ^2^Dipartimento di Psicologia Applicata, FISSPAPadova, Italia

**Keywords:** FSSC-R, FSSC-IT, children's fear, parent's perception, validation study

## Abstract

Studies involving parents' reports about children's fears and multiple informant comparisons are less extended than investigations on children's self-reporting fear schedules. Starting with the Italian version of FSSC-R, the FSSC-IT, the main aims of this study were to adapt a schedule for parents' perception of their children's fear: the FSSC-Parent Perception. Its psychometric properties were examined in a large sample of parents (*N* = 2970) of children aged 8–10 years. Exploratory and confirmatory factorial structures were examined and compared with the Italian children's ones. Mother vs. father, children's gender and school age group effects were analyzed. The confirmatory factor analysis confirmed a four correlated factors solution model (Fear of Danger and Death; Fear of Injury and Animals; Fear of Failure and Criticism; Fear of the unknown and Phobic aspects). Some effects related to child gender, age group, mother vs. father, were found. The FSSC-PP properties supported its use by parents to assess their children's fears. A qualitative analysis of the top 10 fears most endorsed by parents will be presented and compared with children's fears. Clinical implications about the quality of parent-child relationships where discussed, comparing mothers and fathers, and parents' perception about daughters' and sons' most endorsed fears.

## Introduction

According to many authors, normal fears can be considered as an adaptive response, since they motivate attempts to protect from a real or imagined treat (Fisher et al., 2006; Muris, 2007). Childhood fears, as an integral part of normal development, have been widely assessed (Fisher et al., 2006; Verhulst and Van Der Ende, 2006). One of the most used tools to assess normative description of child fears is the Fear Survey Schedule for Children (FSSC; Ollendick, 1983). This schedule and its revised forms (FSSC-II, FSSC-R; Gullone and King, 1997; Gullone et al., 2000; Burnham, 2005; Fisher et al., 2006; Serim-Yıldız and Erdur-Baker, 2013) show the most robust psychometric properties (Svensson and Ost, 1999; Bokhorst et al., 2008; Salcuni et al., 2009; Di Riso et al., 2010; Burkhardt et al., 2012).

Many studies have considered parents as experts for their child's outward behaviors and internal thoughts and emotions, such as fear (Wren et al., 2004; Achenbach, 2006; Weems et al., 2008; De Los Reyes et al., 2010; Muris et al., 2010). Reliance on parental reports, generally regarding anxiety or internalized difficulties, has been based primarily on the assumption that children lack the cognitive sophistication to respond appropriately in a schedule or interview format (Grills and Ollendick, 2003; Achenbach, 2006). Most studies concerning parent-child agreement have been carried out based on structured clinical interviews (for a review see Rapee et al., 1994; Nauta et al., 2004; Achenbach, 2006), then using questionnaire reports (Gullone, 2000; King et al., 2006; Davis et al., 2009; Salcuni et al., 2009; Di Riso et al., 2010; Muris et al., 2010; Serim-Yıldız and Erdur-Baker, 2013). Self-report measures seem to be the most useful and consistent way to focus on fears. Bondy et al. (1985) study was one of the first indicating parents provide an accurate assessment of their children's fears (Scherer and Nakamura, 1968). However, generally, only mothers or parents' (mothers and fathers' together) reports have been considered (Bouldin and Pratt, 1998; Treutler and Epkins, 2003; Muris et al., 2005, 2010; Weems et al., 2008). Only few papers compared the role of parent (mother vs. father) in modeling children's fears, indicating that mothers' fear expression is correlated to children's (Muris et al., 1996; Achenbach, 2006). Only one study has found that fathers reinforce gender stereotypes more than mothers do, in particular for females (Ruble et al., 1988).

Furthermore, to the best of our knowledge, no confirmative studies have been carried out on tools devoted to assess children's fears from parents' perspective, and in particular using the Fear Survey Schedule for Children and its versions. To date, the only one study assessing the structural validity of a parent measure designed to evaluate children's fears was Bouldin and Pratt's (1998) one, who developed the Fear Survey Schedule for Children-II Parent version (FSSC-IIP), to assess fears in preschool children. The authors reported an explorative factor analysis (EFA) with an eight-factor solution including four factors that are conceptually very similar to those obtained by Gullone and King (1997) with the FSSC-II child-version.

In order to fill these gaps, the main aim of the current study was to design and validate a self-report questionnaire suitable for completion by parents assessing their children's fears, starting from the Italian version of the FSSC-R, the FSSC-IT (Salcuni et al., 2009). Thus, the Fear Survey Schedule for Children-Parent Perception (FSSC-PP) would represent the parent version of the FSSC-IT. A majority of the previous confirmative studies on the FSSC-R revealed a four or five factor structure, with only few exceptions (e.g., four-factor structure: Arrindell, 2003; Salcuni et al., 2009; five-factor structure: Ollendick, 1983; Ollendick et al., 1989; Svensson and Ost, 1999; Bokhorst et al., 2008 e.g., seven-factor structure: Mellon et al., 2004). For this reason, for the Italian FSSC-PP a four or five factor structure solution was expected.

Since literature suggests that girls' report significantly higher fears than boys (e.g., Gullone and King, 1997; Westenberg et al., 2004; Muris, 2007) and older children show less fears than younger ones (e.g., Gullone, 2000; King et al., 2006; Davis et al., 2009; Salcuni et al., 2009), studies of children as informants have shown that children's gender and age are associated significantly with different factor scores: girls' fears are always significantly higher than boys' (e.g., Gullone and King, 1997; Westenberg et al., 2004; Muris, 2007) and several studies have found a normative developmental change for which older children reported significantly fewer fears than younger children (e.g., Gullone, 2000; King et al., 2006; Davis et al., 2009; Salcuni et al., 2009). We assumed that parents' perception of girls' fears would be significantly higher than for boys' (Bondy et al., 1985; Silverman and Nelles, 1988; Bouldin and Pratt, 1998; Grills and Ollendick, 2003; De Los Reyes et al., 2010) on all the expected factors. However, due to the narrow age-range of the considered sample (8–10 year olds), no significant age group differences were expected.

Last but not least, in order to further investigate each parent's contribution to children's fears assessment, the present study included mothers, as well as fathers (*N* = 2970) in the same percentage and possible differences within them were evaluated.

## Materials and methods

### Participants

The participants were 1485 Caucasian parental couples (*N* = 2970) of 8–11 year-old school children from mainstream classrooms. The overall response rate of parents who agreed to participate in the study was approximately 78%. Mothers were aged 29–49 years (Mean age 39 years and 7 months, *SD* = 4.37 months) and fathers 28–53 years (Mean age 41 years and 1 month, *SD* = 5.37 months). Parents' socio-economical level, measured by SES (Hollingshead, 1975), was medium. The mean value of educational level of mothers and fathers was 3.78 and 3.91, respectively, (some years of high school) and their occupational level was 4.50 and 6.01, respectively (clerk level). Parents gave their written informed consent to participate in the study and also gave consent for their children. The child group (1392 girls and 1578 boys) comprised 1008 subjects aged 8 years, 870 children aged 9 years and 1092 children aged 10 years.

### Measure

The Italian Fear Survey Schedule for Children (FSSC-IT; Salcuni et al., 2009; Di Riso et al., 2010) is the Italian translation of the FSSC-R (Ollendick, 1983), an 80-item self report, in which no items were changed, except for item 73 where “Russia” was substituted with “Iraq” (Salcuni et al., 2009). The FSSC-IT was back-translated following international guidelines (Van De Vijver and Hambleton, 1996). Previous studies had not used the FSSC-IT with parents. In the present study, the FSSC-IT was distributed to parents and their children. Parents were required to rate their children's level of fear on a three-point scale. Items were scored as: none (1), some (2), and a lot (3), as in the original FSSC-R version (Ollendick, 1983). Italian psychometric studies of the FSSC-IT, and the literature on this tool, show high degrees of internal consistency, test-retest reliability, and construct validity, confirming previous literature findings (Ollendick, 1983; Ollendick et al., 1989, 1991; Mellon et al., 2004; Muris, 2007; Bokhorst et al., 2008; Salcuni et al., 2009; Di Riso et al., 2010). Internal consistency across gender and age group was supported, Cronbach's alpha for the entire schedule was α = 0.96 and α = 0.95–0.96 for boys and girls in each age group.

### Procedures

Prior to conducting the study, approval was obtained from the Local Ethics Committee and informed written consent (Italian law 196/03) was obtained from each participant. Questionnaires were then distributed to 12 primary schools in urban, suburban, and rural areas of Northern Italy. Questionnaires for parents were delivered through the school. Written instructions explained the questionnaire rating system to parents, specifying that there were no right or wrong answers. Parents were asked to indicate how they think their children think and feel.

The original data set included 3126 parents. Statistical analyses, however, were not performed on the part of the sample where values were missing in the schedule. About 2.5% of the parental couples (*n* = 156) were excluded from the research sample because of missing values or when one parent did not answer the questionnaire. For this reason, the sample analyzed included only 2970 parents, half mothers and half fathers. In order to study the structural validity of this instrument, a series of factor analyses were performed. Data were randomly split into two groups, each with approximately 50% of participants. Half the mothers involved in the study were randomly selected as well as half the fathers to make up the first group (calibration sample *N* = 1482; 716 mothers and 766 fathers of 8–10 year-olds: 694 boys and 806 girls); the remaining participants made up the second group (validation sample: *N* = 1488; 769 mothers and 719 fathers of 8–10 year-olds: 791 boys and 679 girls). We assured no more than 2% of the parents were matched rating the same child on each group. Exploratory factor analyses were conducted on the first sample, the calibration sample. Confirmatory factor analyses were then conducted on the validation sample. Groups were balanced for parents' gender [$\chi_{\left(1\right)}^{2}=0.486$, *p* = 0.794] and children's age group [$\chi_{\left(2\right)}^{2}=1.64$, *p* = 0.44].

In order to assess the construct validity of the FSSC-IT on parent's perception of their children's fears, an Exploratory Factor Analysis (EFA) and Confirmatory Factor Analysis (CFA) were used. The goal of the EFA was to reduce the numerous variables (items) measured to fewer more reliable latent constructs, not generally driven by *a priori* theory. The goal of the CFA is to test a theory when the analyst has an adequate rationale regarding the structure of the data. The appropriate use of both methods involves a series of fundamental decisions that directly affect results and interpretations. Exploratory factor analyses (EFA) of the calibration sample were carried out referring to guidelines recommended in Gorsuch (1997) and Fabrigar et al. (1999). A principal component analysis (PCA) with Varimax and Promax rotation was conducted. To determine the number of factors, multiple decision rules were considered (Bentler, 1995; Hu and Bentler, 1999). The screen test (Cattell, 1966), and considerations from previous research were used to determine the number of factors to retain. When interpreting the factors, salience was defined as a loading on a factor ≥0.35. The rule for the number of loadings on each factor was followed (Gorsuch, 1997). Since the factors obtained with the EFA were correlated, a confirmatory factor analysis (CFA) approach was later performed using LISREL8 (Jöreskog and Sörbom, 1996–2001) to determine if the nested model (with correlated factors), derived from the theoretical model found with the Promax EFA, showed a good fit with data. All analyses were performed on the variance-covariance matrices (Cudeck, 1989) and via the maximum likelihood procedure. The item parcels procedure was used to examine the model structure in order to reduce error rates (Little et al., 2002).

The following fit indices were considered by taking into account the rule of thumb cut-off criteria proposed by Schermelleh-Engel et al. (2003): chi-square (χ^2^), a solution fits the data well when χ^2^ is not significant (*p* ≥ 0.05). The Comparative Fit Index (CFI) and the standardized root mean square residual (SRMR) were calculated. CFI should be 0.97 or higher for a good fit; the higher these values, the better the fit (Schermelleh-Engel et al., 2003). The SRMR should be < 0.05 for a good fit (Schermelleh-Engel et al., 2003).

To explore possible influences, an analysis of variance (ANOVA) was performed on the Overall Fear Level score and on the factor scores with parental role (mother vs. father), child's gender and age group as between subjects variables.

Finally, the factor structures identified were compared with those for children (Salcuni et al., 2009) and the qualitative analysis of the 10 most endorsed fears of parents will be presented and compared with those of children (Di Riso et al., 2010).

## Results

The exploratory factor analysis (EFA) was conducted in a two-step process. Step 1: the principal component analysis, conducted on the Pearson intercorrelations, using the initial communality estimates, determined that all values were well below one, indicating the absence of multicollinearity and singularity. In accordance with the Cattell (1966) scree test and previous research, a four-factor structure could be identified, accounting for about 33% of the total variance. Step 2: a principal component analysis was performed with oblique rotation using the Promax method as well as orthogonal rotation with the Varimax method. The two factor structures showed conceptually very similar factors. The Promax rotation reached a simple structure, less items loaded on two factors. The results of these analyses are reported in Table 1.

**Table 1:** **Questionnaire items and corresponding factor loadings from the EFA rotated pattern matrix**.

**Items**	**Factor loadings**			
	**Factor 1**	**Factor 2**	**Factor 3**	**Factor 4**
**FACTOR 1 DEATH AND DANGER**				
41. Being hit by a car or truck	0.833	−0.100	0.079	−0.135
20. Bomb attack, being invaded	0.741	−0.068	−0.079	−0.033
34. Fire, getting burned	0.739	0.080	0.035	−0.168
76. Not being able to breathe	0.724	−0.247	0.003	0.166
58. Falling from high places	0.717	−0.083	0.043	0.088
70. Germs or getting a serious illness	0.707	−0.124	−0.001	0.037
73. Iraq	0.691	−0.190	−0.097	0.082
59. Getting a shock from electricity	0.654	0.018	−0.039	0.026
72. Earthquakes	0.598	0.052	−0.055	0.084
32. Guns	0.585	0.140	−0.077	−0.041
35.Getting a cut or injury	0.545	0.190	0.139	−0.174
26. A burglar breaking into our house	0.530	0.084	0.096	−0.053
56.Deep water or the ocean	0.498	0.017	−0.101	0.190
18. Bears or wolves	0.487	0.387	−0.114	−0.118
10. Getting lost in a strange place	0.480	0.118	0.098	−0.018
07. Sharp objects	0.420	0.228	0.037	−0.052
33. Being in a fight	0.400	0.109	0.116	−0.009
09. Dead people	0.358	0.240	−0.039	0.056
49. Strange-looking people	0.357	0.135	0.156	0.096
**FACTOR 2 INJURIES AND ANIMALS**				
47. Ants or beetles	−0.106	0.775	−0.041	0.007
25. Spiders	−0.077	0.722	−0.041	−0.008
30. Bats or birds	−0.002	0.716	−0.026	0.006
04. Lizards	−0.177	0.697	0.094	−0.073
79. Rats or mice	0.145	0.692	−0.048	−0.093
11. Snakes	0.246	0.643	−0.085	−0.170
78. Worms or snails	−0.092	0.614	−0.005	0.078
21. Getting an injection from a nurse or doctor	0.003	0.496	0.059	−0.003
50. The sight of blood	0.098	0.428	0.143	−0.003
52. Nasty-looking dogs	0.260	0.392	0.029	−0.074
08. Having to go to hospital	0.286	0.357	0.060	0.001
**FACTOR 3 FAILURE AND CRITICISM**				
66. Making mistakes	0.030	−0.059	0.726	−0.006
31. My parents criticizing me	0.047	−0.044	0.684	−0.101
40. Failing a test	−0.149	0.085	0.682	−0.006
29. Getting poor marks for school work	0.175	−0.009	0.665	−0.116
48. Being criticized by others	−0.091	0.128	0.621	0.063
05. Looking foolish	−0.065	0.065	0.573	−0.072
28. Being called unexpectedly by the teacher	0.082	−0.121	0.570	0.080
44. Having my parents argue	0.233	−0.072	0.563	−0.133
64. Getting punished by my father	0.204	−0.072	0.530	−0.090
80. Taking a test	−0.147	0.057	0.526	0.258
24. Being teased	0.023	0.158	0.500	0.012
54. Getting a school report	0.101	−0.160	0.490	0.094
03. Getting punished by mother	0.065	0.028	0.486	−0.078
46. Having to perform in front of others	−0.160	0.079	0.453	0.289
15. Being sent to the head teacher	0.316	−0.088	0.414	−0.127
01. Having to talk to the age group	−0.165	0.047	0.375	0.218
**FACTOR 4 FEAR OF THE UNKNOWN AND PHOBIC ASPECTS**				
74. Lifts and elevators	0.233	−0.175	−0.131	0.590
23. High places like mountains	0.163	−0.005	−0.032	0.498
62. Being alone	0.137	0.061	−0.049	0.493
71. Closed spaces	0.388	−0.068	−0.091	0.484
16. Riding on the train	−0.024	−0.024	−0.032	0.469
02. Riding in the car or bus	−0.053	−0.044	0.002	0.458
75. Dark places	0.115	0.225	−0.051	0.444
60. Going to bed in the dark	0.042	0.280	−0.138	0.432
68. Loud sirens	0.139	0.127	−0.028	0.424
55. Getting a haircut	−0.158	−0.062	−0.048	0.419
36. Being in a big crowd	0.213	−0.040	0.085	0.411
27. Flying in a plane	0.186	−0.015	−0.057	0.410
67. Detective movies	0.017	0.194	−0.058	0.397
17. Being left at home with the baby-sitter	−0.102	0.024	−0.017	0.394
63. Having to wear clothes different from others	−0.061	−0.063	0.219	0.392
42. Having to go to school	−0.161	−0.122	0.210	0.386
65. Having to stay after school	0.197	−0.238	0.201	0.380
69. Doing something new	−0.248	0.080	0.293	0.371
19. Meeting someone for the first time	−0.030	0.155	0.232	0.352
Eigenvalues	16.224	4.137	3.544	2.553
Percentage of variance	20.279	5.171	4.430	3.191

*The number preceding each item represents item position in the sequence of the administered questionnaire*.

The correlations between the factor scores for the Varimax and Promax often showed a medium (0.30–0.40) to high (>0.50) effect size according to Cohen (1988). The Promax EFA structure was chosen for this reason. This solution included 65 items loading the factors (>0.35). The contained factors are conceptually very similar to those found in the literature, so it was decided to retain the original names of factors (Svensson and Ost, 1999; Bokhorst et al., 2008; Salcuni et al., 2009). The criteria used for interpreting the rotated factor pattern was as described above. Factors identified were: Factor 1 Fear of Death and Danger (19 items), Factor 2 Fear of Injury and Animals (11 items), Factor 3 Fear of Failure and Criticism (16 item), Factor 4 Fear of the unknown and Phobic aspects (19 item). Reliabilities of the four dimensions were calculated using Cronbach's alpha. This produced good coefficients (Factor 1 = 0.91; Factor 2 = 0.83; Factor 3 = 0.86; Factor 4 = 0.80). The effect size of the correlation between EFA factors was medium (0.30–0.40) or high (>0.50) according to Cohen (1988).

**Figure 1:**
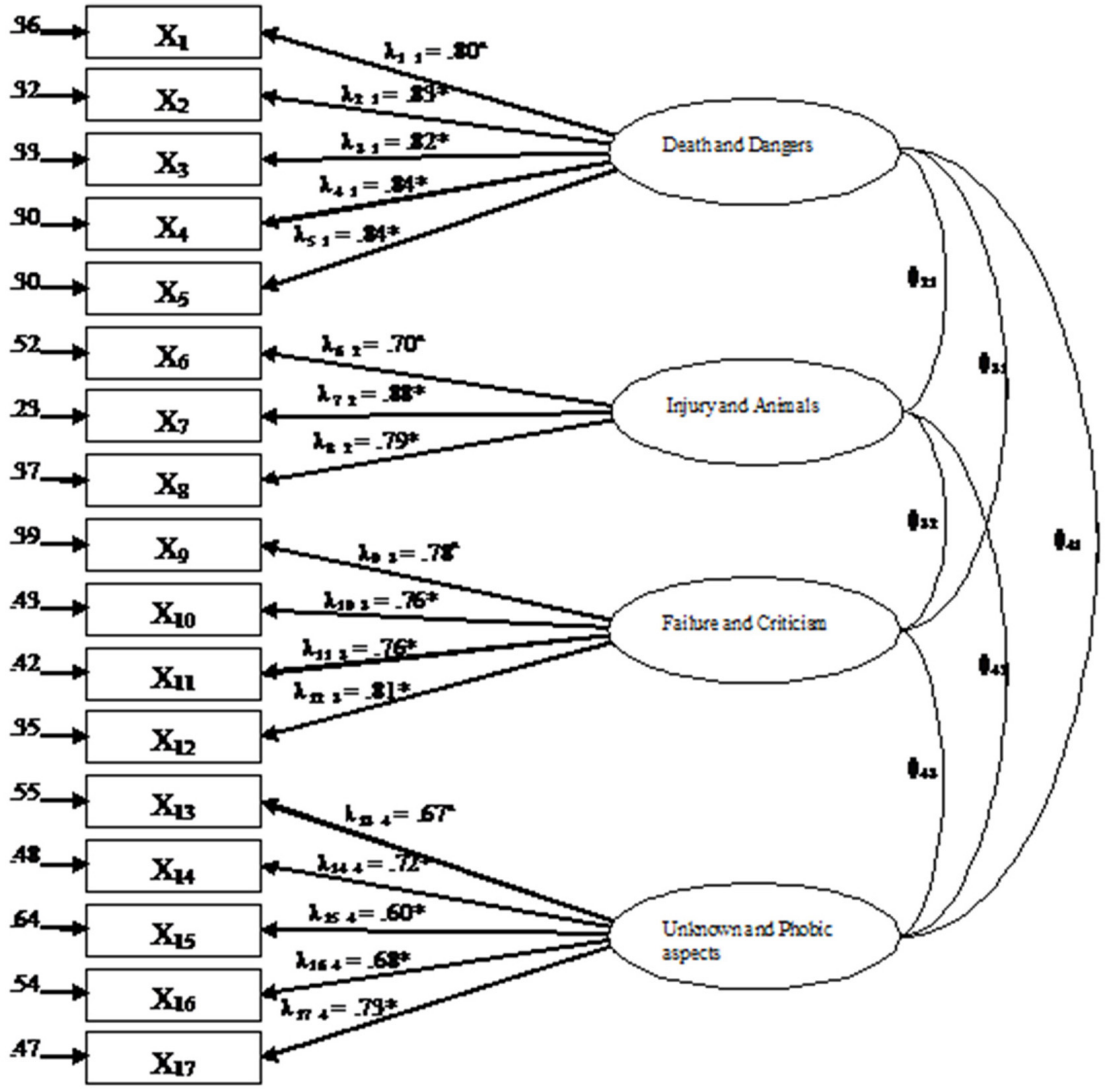
**Standardized path coefficients for CFA model of fear**. Correlations between the four factors are reported in Table 2. Each indicators(X) represent a parcel. ^a^fixed parameter; ^*^p < 0.001.

The goodness of fit of the four-factor model found with EFA was tested in a CFA on the validation sample, using the item parcels procedure. The model evaluated had four latent variables, corresponding to dimensions found in the exploratory Promax factor analysis, and 17 indicators. The indicators were the aggregation of the 65 items in parcels following the procedure of item-to-construct balance suggested by Little et al. (2002). Five parcels were produced for the first and fourth factors, four for the second and three for the third factor. All factors were allowed to correlate and no errors were included in the model. The goodness of fit indices showed that the four-factor model was appropriate for explaining the data. The model with four latent variables showed an excellent fit: $\chi_{\left(113\right)}^{2}=798.73$, *p* = 0.00; CFI = 0.98; SRMR = 0.04. Although the χ^2^ was significant, the other indices satisfied the respective rules of thumb. Factor loadings were all significant (*p* < 0.001) and higher than 0.60. The model produced is presented in Figure 1 with parcel loadings and errors.

**Table 2:** **CFA correlations (coefficients) between the four dimensions of fear**.

**Factor**	**1**	**2**	**3**
1. Death and dangers			
2. Injury and animals	0.63^*^		
3. Failure and criticism	0.46^*^	0.44^*^	
4. The unknown and phobic aspects	0.57^*^	0.58^*^	0.53^*^

* *p < 0.001*.

The CFA model defined a four correlated factor model with 65 items, called the Fear Survey Schedule for Children-Parent Perception (FSSC-PP). A second-order analysis was carried out by examining the correlations among the first-order factors to test the presence of an Overall Fear Level on 65 items for evaluating children's fears. Indices showed that the parcel model fitted the data well: χ${}_{\left(115\right)}^{2}=821.18$, *p* ≤ 0.00; SRMR = 0.05; CFI = 0.98. Factor loadings were all significant (>0.60) as well as a relation of each dimension on the FSSC-PP Overall Fear Level (*p* < 0.001) (range 0.47–0.78).

An analysis of variance (ANOVA) was performed on the total sample, on four-factor scores and the Overall Fear Level on the saturated 65 items, with children's gender and age group, and mother vs. father as between subject variables. The significant results of the ANOVA are summarized in Table 3.

**Table 3:** **Analysis of Variance for principal effects of parental role (mothers vs. fathers), child's gender and age group (*N* = 2970) (interactions are not included)**.

**Factor**	**Mothers vs. fathers**			**Child's gender**			**Child's age group**		
	***F*_(1, 2970)_**	***p***	**η^2^Part**	***F*(1, 2970)**	***p***	**η^2^Part**	***F*_(1, 2970)_**	***p***	**η^2^Part**
1 Death and danger	2.07	0.15	0.001	144.85	0.0001	0.047	3.64	0.026	0.002
2 Injuries and animals	0.001	0.098	0.000	20.13	0.0001	0.007	2.35	0.096	0.002
3 Failure and criticism	4.01	0.045	0.001	309.35	0.0001	0.095	7.98	0.0001	0.004
4 Fear of the unknown and phobic aspects	0.071	0.790	0.000	63.21	0.0001	0.021	2.39	0.092	0.002
Overall fear level	0.013	0.909	0.000	178.68	0.0001	0.057	1.63	0.167	0.001

Parents reported a significantly higher level of fears for girls than for boys in all factors and in Overall Fear Level (Table 4). Although some age differences, some mother vs. father differences and some interactions between variables were significant, a partial eta-square estimate was not within the significant range (1–5% effect sizes). Table 4 reported means and standard deviations for children according to gender.

**Table 4:** **Boys' and girls' means and standard deviations on FSSC-PP factors and overall fears level**.

**Factor**	**Gender**			
	**Boys**		**Girls**	
	**(*N* = 1578)**		**(*N* = 1392)**	
	***M***	***SD***	***M***	***SD***
1 Death and danger	17.95	8.72	21.63	8.54
2 Injuries and animals	11.72	5.96	12.66	5.54
3 Failure and criticism	7.48	4.37	10.37	4.77
4 Fear of the unknown and phobic aspects	5.98	4.71	7.40	5.13
Overall Fear Level	43.13	18.84	52.06	18.39

In the present study, using the FSSC-IT to assess parents' perceptions of their children's fears, a four-factor solution was found. The four factors obtained were similar to the most frequent factors found in the literature (Ollendick, 1983; Ollendick et al., 1989, 1991; Svensson and Ost, 1999; Bokhorst et al., 2008; Salcuni et al., 2009). Despite these similarities, the factors obtained saturated a different number of items and presented a different rank order, in particular, if compared with other Italian work with the FSSC-IT (Salcuni et al., 2009).

A close comparison between the FSSC-IT structure analysis (Salcuni et al., 2009; Di Riso et al., 2010) and the FSSC-PP showed a factorial structure similar, but not the same as, that regarding Italian children (Salcuni et al., 2009). First, the CFA model considered 65 items, instead of the original 80 (Ollendick, 1983) or 60 (Salcuni et al., 2009), which were highly loaded on the exploratory factor analyses of the four factors. In this paper, we considered a 0.35 saturation instead of 0.40 (Salcuni et al., 2009). Comparing the item distribution in the FSSC-PP and FSSC-IT per factor, with 36 items overlapping, some differences were also found in rank order of saturations. To determine whether the FSSC-PP was measuring similar constructs found in Italian children (Salcuni et al., 2009; Di Riso et al., 2010), coefficients of congruence (Robert and Escoufier, 1976) between the factors obtained through principal component analysis with varimax rotation in the present sample of scores, and those reported by Salcuni et al. (2009) were reported (Table 5).

**Table 5:** **Coefficient of congruence values comparing the FSSC-PP and the FSSC-IT**.

**Factor FSSC-PP**	**Factor FSSC-IT (Salcuni et al., 2009)**	**Coefficients of congruence**
I (Death and danger)	I (Death and danger)	0.981
II (Injuries and animals)	II (Injuries and animals)	0.989
III (Failure and criticism)	III (Failure and criticism)	0.994
IV (Fear of the unknown and phobic aspects)	IV (Fear of the unknown)	0.864

The first 10 fear items that parents most frequently endorsed with “a lot” (3) are presented in Table 6, both for the overall sample and separated for mothers and fathers. Items are listed in decreasing order according to the percentage of overall sample.

**Table 6:** **Most frequently endorsed fears with greatest intensity for overall sample (*N* = 2970), mothers (*N* = 1485) and fathers (*N* = 1485)**.

**Item number**	**Loading on factor**	**Overall sample**		**Mothers**		**Fathers**	
		**Freq**.	%	**Freq**.	%	**Freq**.	%
26. A burglar breaking into your house	1	1693	57	880	59.3	813	54.7
10. Getting lost in a strange place	1	1483	49.9	774	52.1	709	47.7
41. Being hit by a car or truck	1	1409	47.4	723	48.7	686	46.2
34. Fire, getting burned	1	1382	46.5	688	46.3	694	46.7
20. Not being able to breathe	1	1256	42.3	653	44	603	40.6
11. Snakes	2	1090	36.7	526	35.4	564	38
52. Nasty-looking dogs	2	973	32.8	482	32.5	491	33.1
72. Earthquakes	1	978	32.9	479	32.3	499	33.6
8. Having to go to hospital	2	948	31.9	498	33.5	450	30.3
15. Being sent to the principal	3–1	931	31.3	492	33.1	439	29.6

The top 10 fears are quite common in mothers and fathers, with minimal differences in rank order. Most of them loaded in Factor 1 Fear of Danger and Death, and 3 items on Factor 2 Fear of Injuries and Animals. Item 15 was an exception, loading both in Factors 1 and 3 for FSSC-PP, as in FSSC-IT (Salcuni et al., 2009; Di Riso et al., 2010). The comparison on item saturation in the two samples reached a 0.987, very high congruence coefficient (Robert and Escoufier, 1976).

A comparison between the distribution of the most endorsed fears items in FSSC-PP and in that for children, in particular the FSSC-IT (Di Riso et al., 2010), showed that the distribution of the top 10 items was similar to many previous studies with children, in various countries (Ollendick et al., 1991; Varela et al., 2008).

Compared with children, parents presented, as expected, a generally lower percentage of level 3 in their scoring. According to the literature, children most frequently indicate “a lot” in their evaluation of fear (Ollendick et al., 1991; Varela et al., 2008). In particular, a previous study with Italian children (Di Riso et al., 2010) showed the frequency of score 3 ranged from 61.4 to 41%, in contrast with parents for whom the range was 57 to 31.3%. Seven of 10 items are the same in both the children's and parents' questionnaires, although the rank order did not correspond entirely. Three of the items which parents scored as “a lot” when considering their children's fears, did not appear in the 10 most-endorsed items for children: 2 were for Factor 2 in FSSC-PP but Factor 1 of FSSC-IT (11, Snakes; 52, Nasty-looking dogs) and one was not included in any factor (8, Having to go to hospital). The 3 items children considered particularly fearful, which parents did not (Di Riso et al., 2010), belonged to Factor 1 in FSSC-IT and in FSSC-PP (20, Bomb attacks, being invaded; 58, Falling from high places; and 73, Iraq).

## Discussion

The present study examined a factorial structure and the psychometric properties of the Fear Survey Schedule for Child Parent Perception (FSSC-PP) in a large non-clinical sample of Italian parents of children, aged between 8 and 10 years. This is the first study that has involved both Italian parents of children in this specific age group.

The FSSC-PP questionnaire has 65 items, compared with 80 items in the original FSSC-R (Ollendick, 1983), and could be more suitable for completion by parents when assessing their children's fears. Results from the CFA report a model with four correlated factors, very similar to that found in the literature for children (Ollendick, 1983; Ollendick et al., 1989; Svensson and Ost, 1999; Muris and Ollendick, 2002; Bokhorst et al., 2008; Salcuni et al., 2009). The four-factor item distribution (65 items with 0.35 or higher saturation) was substantially the same as reported in the literature (Muris and Ollendick, 2002; Muris, 2007). Salcuni et al. (2009) using the FSSC-IT, identified a five-factor structure, although only four factors could be interpreted since the fifth was loaded by few items, most of which also loaded on other factors. In the present study, the FSSC-PP yielded four somewhat similar, but not equivalent factors. Although the factors presented the same names, with the exception of the fourth, they each saturated a different number of items, and presented some differences in rank distribution. The coefficients of congruence comparing the pairs of factors were calculated and they met the a priori criterion of 0.90 for all Factors, but the Factor 4. This finding suggests the factorial structure is appropriate for the scores of Italian parents.

Correlations between the four dimensions also suggested the existence of a high-order anxiety factor, that is a single and multifaceted dimension of fear that might be useful for both research and clinical purposes (Muris, 2007). Cronbach's alpha ranged from 0.91 to 0.80, and this supports the use of the Overall Fear Level score and factors in further investigations and assessment of fears in children (Muris and Ollendick, 2002).

In terms of gender differences, as expected, girls were always perceived as significantly more fearful than boys (Mellon et al., 2004; Muris, 2007; Bokhorst et al., 2008; Di Riso et al., 2010). Age group differences, parental role (mother vs. father) and interaction between independent variables, although significant, were not robust. It should be noted, however, that the study only considered a narrow age band, whereas studies which have found age differences have included a larger age sampling, with adolescents as well as younger children. The effect of gender was significant and strong (η^2^ > 0.005) for all the Factors and for the overall fears level score. As expected child's gender also played a fundamental role in parental perception of children's fears (Bondy et al., 1985; Silverman and Nelles, 1988) and parental perception of girls' fears was higher than for boys' ones. One possible explanation for this finding may be found in gender differences, which appear to be related to parental rearing practices that differ for girls and boys, as well as the willingness of girls to report fears more readily than boys (Ginsburg and Silverman, 2000; Muris et al., 2005). In particular, we found both mothers and fathers contribute to the gender stereotyping of their children for each kind of fear. It seems that mother and fathers, equally, send and receive subtle messages regarding gender, and about what is expected and accepted for each gender. These messages are then internalized by the developing child (Arliss, 1991). Gender role stereotypes are well established in early childhood and messages about what is appropriate are so strong, that even when children are exposed to different attitudes and experiences, they revert to stereotyped choices. Gender stereotypes and biases seem to occur within the family setting, influencing parents both overtly and covertly in their representation of children's worries and fears.

In a comparison of parents' and children's questionnaires, differences in the top 10 fearful items showed parents' concerns were mostly focused on real everyday life fearful events, such as animals or injuries, even children were mostly impressed by general external and violent events, connected maybe with television news, such as war, bombing or accidental falls from high places. Moreover, top 10 most endorsed fears in children's reports belonged to first factor of FSSC-IT, which included only death and danger fears: instead it was not for parents whom top 10 includes fears of animals and of social situation.

This finding could be explained as a normal difference in an adult's vs. child's way of categorizing events. Parents (asked to score as if they were their children) considered their children to be more able than they were, to differentiate between real and immediate dangers instead of imaginary and distant dangers. This finding was in line with the literature which documents the not-perfect parent-child agreement on multiple informant studies (Bondy et al., 1985; Cole et al., 2000; Grills and Ollendick, 2003; Bögel and Van Melik, 2004; Foley et al., 2004; Nauta et al., 2004; Wren et al., 2004; Achenbach, 2006; De Los Reyes et al., 2010).

Clinical implications of this study focused on the possibility to assess the gap between children fears and parents' perception of their children's fears, considering first of all the importance of parental alliance to any kind of treatment or intervention that professionals might start with patients in developmental age (Gardner and Shaw, 2008); any kind of intervention on children “requires assessment of the presenting problems in the context of family and caregiver influences, as well as the child's development and physical health” (Gardner and Shaw, 2008, p. 887). Being aware of the level and the quality of their child's emotional problem—such as fear or phobia—could be useful to help parents to understand the gap between their parental perception of child's fear and child's fear evaluation. Parenting intervention is generally the treatment of choice for any kind of developmental problem: increasing in parents their comprehension of children point of view about their fears, and showing how parents themselves tends to considered their children to be more able than they were, to differentiate between real and immediate dangers instead of imaginary and distant dangers, can be the base of therapeutic alliance with parents. This could be considered the starting point to support both clinical compliance and parenting strategies in helping parents to cope with particularly fearful children: clinical fears schedule for children (FSSC-IT) and for parents (FSSC-PP) might be compared and proposed to the attention of parents. Sharing diagnosis and assessment data and making sense of a problem (Finn, 2007), reduce parental blame and guilt (Gardner and Shaw, 2008) and improve the effectiveness of parenting interventions for possible emotional or behavioral problems (Turner et al., 1994; Kerwin, 1999; Turner and Sanders, 2006). Parental involvement in cognitive-behavioral interventions (Ollendick and King, 1998) as well as in psychodynamic approach (Finn, 2007; Tharinger et al., 2009) is useful with children, especially where parents are very anxious (Barrett et al., 1996), and may be helpful, for example, starting from FSSC results and more endorsed fears, using pictures and drawing techniques (Hirshfeld-Becker and Biederman, 2002) and sharing the assessment videos (Finn, 2007) to help children and their parents with discussions about emotions and fears.

This paper leaves many questions to be answered in future studies. Some limits of the present study must be summarized. The effect of variables in socio-economical status were not controlled, and no effect size was calculated. The use of fear measures must rely on a proven capacity of the instruments to measure factors that are not dependent on cultural or linguistic contexts. An important prerequisite for carrying out confirmatory factor analyses across national samples is the demonstration of the cross-national stability of the dimensional of fears involved. This study does not make a contribution to cross-cultural psychology, confirmatory factor analyses need to be carried out with different samples to verify the present model.

Furthermore this study only involved parents of 8–10 year olds. Samples of Italian early adolescents or adolescents should be used in future studies. This study also involved a community sample so the findings cannot be generalized to clinical samples.

Multiple informant agreement with questionnaires, in particular, has been studied less, even if it is suggested that pulling together different sources of information derived from questionnaires could give a more reliable and valid source of information. Most of the studies have been carried out on informants' agreement with the factor structure of parents' and children's questionnaires, especially on anxiety symptoms (Cole et al., 2000; Grills and Ollendick, 2003; Achenbach, 2006), and different factor structures were found. It could be very interesting to focus future studies on parent-child comparison in a multiple-informant prospective on fears perception.

Finally, an important field to explore in order to explain some differences between informants' data, could be the correlation between parents' fears and parents' perception of their children's fears. In order to explain these results further, future studies need to focus on other moderating variables, such as parental fears linked with children's fears, parental attribution styles, gender role orientation evaluation, and others. Future research into the relationship between normal fear experiences and other developmental experiences (e.g., parenting styles and family experiences) as well as other individual difference variables is required.

In sum, this paper is a first attempt to assess the factorial structure, reliability, and validity of the FSSC-PP when administered to a large non-clinical group of Italian parents. The results supported the FSSC-PP general model with four correlated factors evidencing similarities with previous studies of children's FSSC-IT (Salcuni et al., 2009; Di Riso et al., 2010) and FSSC-R (Bokhorst et al., 2008). In conclusion this study highlights the importance of involving parents as informants in children's fear assessment, to contribute to an early screening of normal fears and prevent psychopathological risk.

### Conflict of interest statement

The authors declare that the research was conducted in the absence of any commercial or financial relationships that could be construed as a potential conflict of interest.
